# Welfare Assessment and Identification of the Associated Risk Factors Compromising the Welfare of Working Donkeys (*Equus asinus*) in Egyptian Brick Kilns

**DOI:** 10.3390/ani10091611

**Published:** 2020-09-09

**Authors:** Shaaban F. Farhat, Amy K. McLean, Hamdy F. F. Mahmoud

**Affiliations:** 1 Egyptian Society for Protection and Welfare of Working Animal (ESPWWA), Cairo 11865, Egypt; 2 Department of Animal Science, University of California Davis, Davis, CA 95617, USA; 3 World Donkey Breed Project, University of Cordoba, 14014 Cordoba, Spain; 4 Department of Statistics, Virginia Polytechnic Institute and State University, Blacksburg, VA 24061, USA; ehamdy@vt.edu; 5 Department of Statistics, Mathematics, and Insurance, Faculty of Commerce, Assiut University, Assiut 71515, Egypt

**Keywords:** Donkeys, welfare, behavior, working equids, brick kilns

## Abstract

**Simple Summary:**

Working donkeys suffer from many welfare challenges associated with, for example, physical health, poor living conditions, and unfair treatment. The aim of this study is to assess the welfare of working donkeys in the El-Saf brick kilns, identifying the health risk factors, establishing welfare regulations, enacting legislation, and implementing welfare strategies aimed at improving the quality of life of donkeys and owners within communities. The study found that working donkeys in Egypt suffer from many types of wounds associated with parts of the harness, such as the saddle, breeching, and neck collar, and with excessive force/beating, the shaft of the cart, and improper tethering. They often live in unhealthy housing situations, and a high percentage suffer from aggressive behavior. The study found an association between these health risks, behavioral parameters, and body condition in Egyptian working donkeys. Body condition was affected by multiple factors, including the number of hours worked/day, the number of donkeys/kilns, the distance from loading to unloading bricks in an oven, and the amount of concentrated food/donkey.

**Abstract:**

Donkeys are a cornerstone in human existence, having played an important role throughout history in different economic activities, such as working in brick kilns in Egypt. This study was conducted from January 2017 to the end of April 2017 in the El-Saf brick kilns, which are located to the south of the Giza Governorate and 57 Km away from Cairo. Physical clinical health and behavior data were collected from 179 donkeys spanning over a random sample of 20 brick kilns selected from the El-Saf brick kilns. Behavioral, physical health, harness, and environmental parameters were assessed and recorded. The study found that 80 ± 3% (n = 179) of kiln donkeys have some type of wound, and the most serious wound is a beating wound (49 ± 3.7%), which is caused by drivers hitting the donkeys. The drivers are mostly children, who have insufficient knowledge, skills, and attitudes to effectively communicate with their donkeys and no motivation to enhance the welfare of these equids. Other wounds are related to the harness, such as the breeching (10 ± 2.2%), saddle (43 ± 3.7%), neck collar (40 ± 3.6%), and shaft of the cart (12 ± 2.4%). A poor body condition was seen in 56 ± 3.7% of kiln donkeys. A correlation in terms of the prevalence of wounds was found between the body condition (*p*-value < 0.01) and/or cleanliness of the harness. There was a negative association between the body condition and wound prevalence in brick kilns (Pearson coefficient of correlation −0.71). The physical enviromental factors that affect the body condition of working donkeys are the working hours of donkeys/day, the number of donkeys in a kiln, the distance from loading to the oven, and the concentrated food/donkey (*p*-value < 0.01). These three variables can explain 78.85% of the variability in body conditions based on a 1–5 scale. In addition to health parameters, behavior parameters, such as the donkeys’ general attitude, reaction to observers, and chin contact are associated with the body condition (*p*-value < 0.01). As a consequence, it is important for the owners of working donkeys to pay attention to their body condition in order to avoid compromising their body condition and welfare.

## 1. Introduction

Donkeys are a cornerstone in human existence and have played an important role throughout much history in different economic activities. The world donkey population is estimated to be about 43 million, with approximately 95% of them used for work in developing countries carrying out domestic activities, as well as tasks relating to agriculture, transport, and different industries (e.g., construction) [1,2,3]. It is not uncommon to find working donkeys suffering from many welfare-related problems, including wounds, poor body condition, respiratory diseases, parasites, a poor dental condition, and lameness [3,4,5,6]. Welfare problems do not stop with physical ailments; many working donkeys experience compromised mental states, such as a fear of humans and even depression [4]. Poor welfare is commonly associated with working donkeys in Africa and the Middle East. Their poor welfare has been linked to both harsh working conditions [3,5] and to handlers with insufficient knowledge of general husbandry and properly caring for working donkeys, such as wound management, harness care and fitting, watering, nutritional requirements, appropriate shelter arrangements, and veterinary services [6,7,8,9].

Approximately 3.7% of the world donkey population is found in Egypt, with close to 1.6 million animals [10]. Donkeys have a long history in Egypt, having been first domesticated there around 3000–4000BC [11]. Recently, the increasing human population has caused an increase in construction activities, including the building of new cities, and this has increased the demand for bricks. Consequently, there has been an increase in the demand for working donkeys in Egypt. Despite the increase of mechanization in brick kilns, donkeys are still well deserving of the name the “beasts of burden”, and are heavily relied upon for production. In the El-Saf brick kilns, where the Egyptian Society for the Protection and Welfare of Working Animals (ESPWWA) is located, the donkey is the preferred working equid. Donkeys are responsible for transporting green dried bricks from the loading area to the ovens inside by pulling overloaded carts, which are driven by children. Donkeys working in these brick kilns face multiple challenges. They often receive little nutrition and veterinary care, and they are often over-worked, overloaded, and wear ill-fitted, poorly designed harnesses [4]. These deficiencies in care lead to many welfare problems, including harness-induced injuries, dehydration, wounds from beating, lameness, and other health problems [12]. There is no training for donkeys in performing such hard work. The average load of bricks weighs 6 to 12 times the body weight of a donkey found working in the kilns. Apart from pulling heavy loads, the donkeys work in a very harsh environment for long hours, pulling loads on difficult and uneven terrain and being exposed to extreme temperatures from the heat radiating from the ovens. Furthermore, the handlers are young children or adolescents, who have little knowledge of how to properly work, care for, and communicate with donkeys. Thus, the donkeys are subjected to many conditions that compromise their welfare. Despite their invaluable contribution to brick kilns and to sustaining over 250 workers/brick kiln, donkeys are the most neglected part of the industry, with brick kiln owners considering donkeys as machines with a low economic value. The welfare of working donkeys is very important not only for the health and survival of those animals, but also for the livelihood of the people who depend on them [9]. A welfare assessment of working equids is crucial for establishing welfare regulations, enacting legislation, and implementing welfare strategies aimed at improving the quality of life of owners and equids within communities [13]. The use of a combination of physical, behavioral, and mental parameters has been commonly used in the welfare assessment of working equids [5,14,15,16,17]. The ESPWWA faces many challenges in assessing the welfare of donkeys in brick kilns, including the fact that the work in brick kilns is temporary, and there is a dynamic movement of handlers from kilns. This variation of the welfare of donkeys in brick kilns may help us to understand the risk factors, including management practices that can lead to the compromise or improvement of donkey welfare. Studies focused on identifying the conditions that have a severe and negative impact on welfare, along with research that focuses on how to successfully implement changes that improve donkey welfare, are important. However, the current research in both of these areas is limited [18]. The aim of this study is to assess the welfare of working donkeys in the El-Saf brick kilns and identify the associated risk factors that may compromise the welfare of working donkeys in many Egyptian brick kilns.

## 2. Materials and Methods

### 2.1. Study Area

The study was conducted from January 2017 to the end of April 2017 in the El-Saf brick kilns, south of Giza Governorate, 57 Km from Cairo, Egypt. There are 250 brick kilns in El-Saf, with 120 operating kilns. Twenty kilns (16.6% of all kilns) were randomly selected from the operating kilns for the study.

The operating kilns are home to 1350 male donkeys and 55 mules, and 179 donkeys from this population were surveyed for this study. No female donkeys work in the brick kilns. The 20 selected kilns had no mules, so the random sample in this study consisted of 179 male donkeys.

### 2.2. Behavior Parameter Examination

Physical, clinical, and behavioral data were collected from the 179 donkeys in the random sample of 20 brick kilns selected from the El-Saf brick kilns. The behavior parameters were measured first, as soon as the donkey had completed its work [19]. The following measurements were assessed and recorded: general behavior (alert, apathetic, or depressed), response to an observer (unfamiliar person), and acceptance of chin contact. The sequence and procedure for taking the behavior measurements was as follows: to assess the alertness of the donkey (alert, apathetic, or depressed), and to observe the donkey from a distance of at least 3 m and for up to 10 s. Then, the observer walked slowly at an angle of 30° (based on visual observation) toward the head of the donkey and stopped approximately 30 cm from the head of the donkey to assess the response of the donkey at the moment that the observer stopped. Next, the donkey’s reactions that fall within the following categories were recorded: discovering, not moving, avoiding, running away, and showing an aggressive reaction. Lastly, the observer put his hand very gently under the donkey’s chin, enough to take some weight but not so much as to lift the head. If the donkey took his head away from the hand, the observer would not pursue it and recorded the acceptance of chin contact (acceptance or non-acceptance). After initiating the physical contact, the observer started the physical and health examination.

### 2.3. Physical Health Parameter Examination

**Figure 1:**
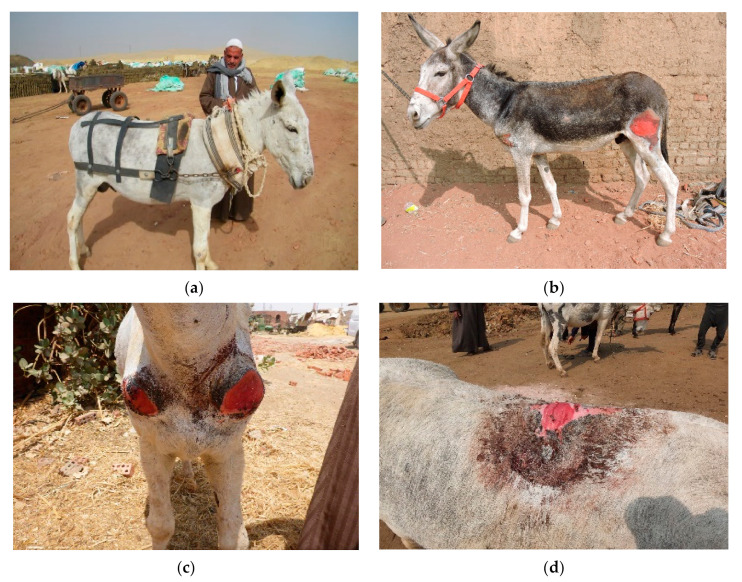
This figure shows a donkey with a full harness and different types of wounds caused by the harness: (**a**) a full harness setting, (**b**) a breaching wound, (**c**) a neck collar wound, and (**d**) a saddle wound.

**Figure 2:**
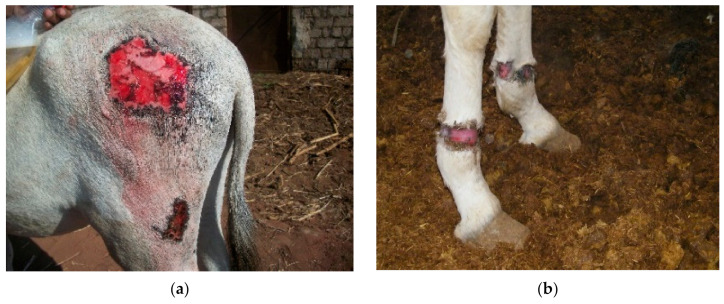

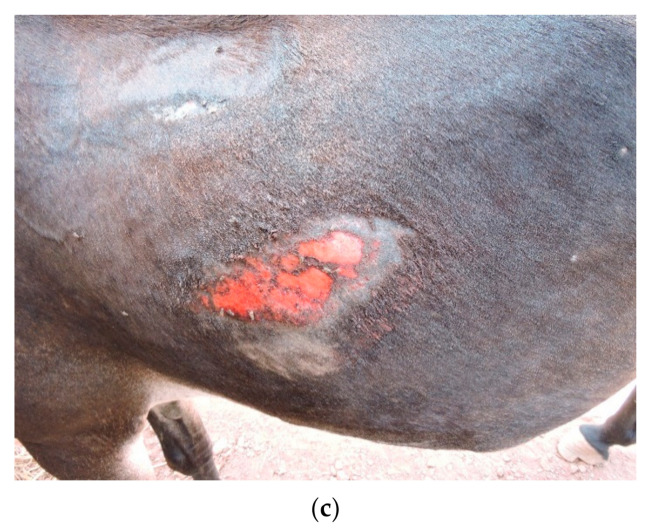
(**a**) A wound from excessive force/beating, (**b**) a limb wound from improper tethering, and (**c**) a shaft wound from the cart.

**Figure 3:**
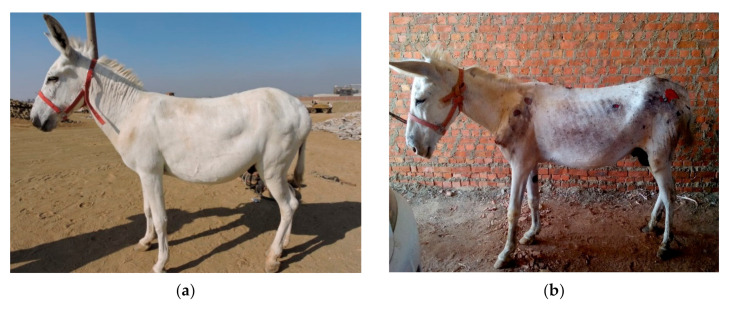
(**a**) Body condition was assessed using a scale of 1 (poor) to 5 (obese). The donkey in (**a**) would be considered to have a good body condition (3) and no wounds, and the donkey in (**b**) would be considered to have a poor body condition (2) and multiple lesions from the harness and excessive force.

The physical examination started from the head and progressed toward the rear of the working donkey. Any abnormalities or skin lesions were recorded. In addition to the number of wounds, the area of the wounds (multiplying the width by the length) were estimated as well. The wounds were classified into two categories, harness-induced wounds, and excessive force/beating wounds. The harness wounds were recorded and related to the anatomical part of the donkey’s body and the part of the harness where the lesions were found, for example, lesions on the back caused by the saddle; wounds on the shoulders or top of the neck from the neck collar; wounds on the chest or sides from the shaft of the cart; and lesions on the hind legs or hips from the breeching. Lesions and wounds inflicted by the driver from excessive force were described as beating wounds. Figure 1 shows how the harness should fit (Figure 1a), and the other images (Figure 1b–d) display different types of wounds related to ill-fitting harnesses. Figure 2a–c shows wounds caused by excessive force and beating, limb wounds from improper tethering, and shaft wounds caused by the cart. Other physical parameters were measured, such as the age, estimated from the mandibular incisor occlusal table appearance [20]. The body condition was recorded using a scale from 1–5, where 1 stands for poor, and 5 stands for obese (1–5) [21]. The body weight was measured by measuring the body height (from the ground to the top of the withers) and by measuring the circumference of the heart girth. Then, the results were plotted on a nomogram [22]. Figure 3 shows two donkeys, one with a good body condition (Figure 3a) and a working donkey with a poor body condition (Figure 3b).

### 2.4. Physical Environmental Parameters Assessment

**Figure 4:**
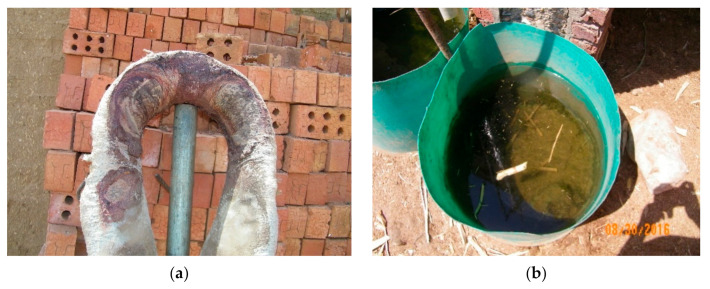
Examples of parameters compromising working donkey welfare including (**a**) a dirty neck collar, and (**b**) unclean water.

**Figure 5:**
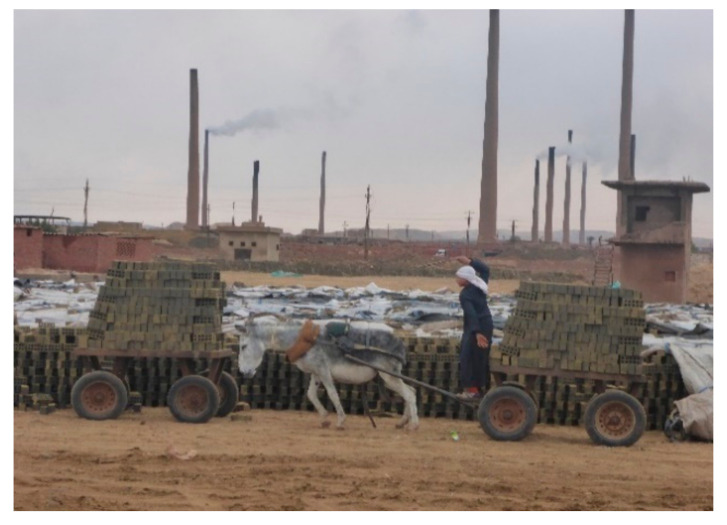
A cart loaded with brick cargo; the driver hits the donkey on its rump.

In addition to physical and behavioral parameters, environmental parameters were assessed and recorded, such as the harness, cart, housing, watering, feeding, roads, and the load of bricks. During the period when the donkey was working, the following parameters were assessed: time working, harness condition, harness cleanliness, and the fitness of the harness. The condition of the harness was scored from 1 (very bad) to 5 (very good), as described in Table 1. The cleanliness of the harness was assessed from 1 (bad) to 3 (good) (Table 2). Figure 4a shows an example of an unacceptable or bad neck collar. The fitting of the hitching point, neck collar, and breeching were assessed and noted as either fitting or not fitting the animal. The cart condition was assessed by giving an overall rating to the cart’s wheel bearings, tires, and shafts. The cart condition was scored from 1 (bad) to 3 (good), as described in Table 3. The road from the loading area to the oven was evaluated according to the amount of debris from bricks. A bad rating was given if the path had a lot of broken bricks that hindered the movement of the carts; a fair rating was given if a small amount of broken bricks were found in the path; and the path was considered to be good if the path was free of brick debris. Roads were assessed based on their slope. A bad rating was applied if there was a negative slope (traveling to or from the kiln); it was fair if a slope was found on part of the path; and it was good if a slope toward the oven was present. The water was assessed based on several parameters, such as clean or unclean, taste (salty or sweet). If the water was found to be salty or unclean, it was considered to be bad. Water found to be clean and sweet was scored as a good water source. Figure 4b shows unclean water. Donkey housing was assessed in terms of the floor condition (bad if it is muddy, dirty with urine and fecal matter; good if it is clean and dry) and ventilation (bad if there are not enough windows; good if there are enough windows). Brick loads were measured three times, first at the beginning of the workday, two hours later, and before the end of the workday. The average brick load was recorded. Figure 5 shows a cart loaded with bricks. Other parameters were assessed, such as the hours worked/day/kiln, the number of ovens in a kiln, the distance between the loading point and the oven, and the average amount of concentrate food provided for each donkey.

### 2.5. Statistical Analysis

To assess the welfare and identify the risk factors compromising the welfare of donkeys, as well as explore the association between these factors, different types of analyses were performed. Descriptive analyses were performed with numerical variables, such as the percentage, means, standard errors, median, maximum, and minimum. A chi-square test was performed to explore the association between variables and factors. T-tests were performed to compare donkeys with wounds to donkeys without wounds and considered to be healthy. ANOVA and Tuckey pairwise multiple comparisons were performed to compare the impact of body condition related to different types of wounds and/or behavioral parameters, and to detect groups that are statistically different in terms of body condition. Multiple linear regression was used to find the factors that impacted body condition. Two types of statistical software were used to analyze and graph the data. Minitab version 19 (Minitab, Inc., State College, PA, USA) and R version 3.6.3 (Free Software Foundation, Inc., Boston, MA, USA).

## 3. Results

### 3.1. Physical Health Parameters

The average of the numerical parameters was calculated and is displayed as the mean ± standard error. The average age of the donkeys in this study was 13.23 ± 0.32 years, and their body condition was 2.41 ± 0.06. The incidence of different types of wounds was calculated and is displayed as the percentage ± standard error. The study found that 80 ± 3% of kiln donkeys had some type of wounds, and 49 ± 3.7% had lesions or wounds caused by excessive force or beating. For the harness-related wounds, 43 ± 3.7% of kiln donkeys had wounds caused by the saddle, and 40 ± 3.6% had wounds in the neck region from the collar. These last two wounds were the result of unfit, dirty harnesses and the poor body condition of the working donkeys, as we found in this study. Wounds were found on 16 ± 2.7% of the donkeys’ limbs, and of these wounds, 10 ± 2.2% were caused by the breeching, and 12 ± 2.4% were caused by the shaft of the cart. Other wounds represented 13.5 ± 0.82%, which were found on the donkeys working in the kilns.

For the wound area, Table 4 shows a numerical summary of the area of the different wound types in cm^2^ as the mean ± standard error. The serious wounds included both the wounds/lesions caused by the shaft (89.60 ± 31.2 cm^2^) and breeching wounds (77.80 ± 40.3 cm^2^). However, few donkeys had wounds that covered large areas; therefore, the median measure was also reported. The highest median was found for beating wounds (30 cm^2^), followed by breeching wounds (19 cm^2^) and shaft wounds (16 cm^2^).

The chi-square test was used to test the association between the different types of wounds and the cleanliness, condition, and fit of the harness. We found that neck collar, saddle, and breeching wounds were associated with the level of cleanliness (*p* < 0.01). The neck collar wounds were associated with the condition and fit (*p* < 0.01). For the association between the most serious wounds, i.e., beating wounds, and other types of wounds, the chi-square test found a correlation between beating wounds and saddle and breeching wounds (*p* < 0.01). In addition, anssociation was found between wounds caused by beating and the overall condition of the harness including fit, condition, and cleanliness (*p* < 0.01). A relationship was found between the cleanliness of the saddle and breeching (*p*< 0.01) and the fit of the neck collar.

**Figure 6:**
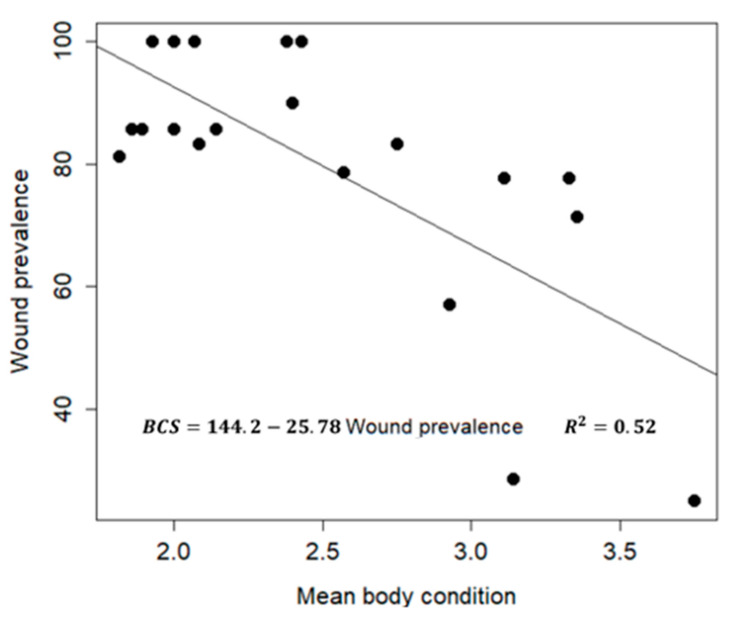
A scatter plot of the mean body condition and wound prevalence (%) in the 20 brick kilns selected, along with a regression line.

The results found that 56 ± 3.7% of the donkeys had body condition scores of 2 or less. A t-test was used to study the association between body condition and skin wounds. A significant difference (*p* < 0.01) was found in body condition, where injured donkeys had body condition scores of 2.3 ± 0.06, and healthy donkeys had a higher BCS (Body Condition Score) (2.9 ± 0.17). This suggests that there was an association between body condition and injuries or wounds, which means that donkeys received better treatment, healthier food, and improved care, and they had an overall better body condition and fewer injuries. For each type of wound, the body condition of the donkeys was found to be as follows: donkeys with saddle wounds (2.22 ± 0.08) and without saddle wounds (2.56 ± 0.9); donkeys with limb wounds (1.98 ± 0.11) and without limb wounds (2.49 ± 0.07); and donkeys with neck collar wounds (2.17 ± 0.08) and without collar wounds (2.57 ± 0.08). The scatter plot in Figure 6 shows that there was a negative association between the two parameters, wound prevalence and BCS. Additionally, the Pearson coefficient of correlation was −0.71 between body condition and wound prevalence in brick kilns. The regression equation was BSC = 144.2 − 25.78 (wound prevalence). The *p*-value of the intercept and slope are 1.8e-08 and 0.0003, respectively, and the coefficient of determination *R^2^* = 0.52.

### 3.2. Behavioral Parameter Assesment

**Figure 7:**
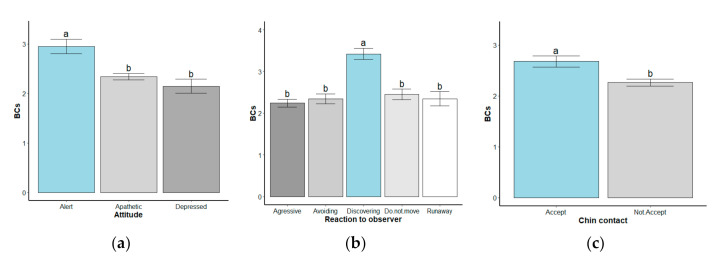
Error bars for the statuses of the behavior parameters: (**a**) the mean body condition of alert, apathetic, and depressed kiln donkeys, along with the mean ± standard error of the mean; (**b**) the mean body condition of donkeys that were aggressive and avoidant and that reacted to the observer by discovering, not moving, and running away, along with the mean ± standard error of the mean; (**c**) the mean body condition of donkeys who accepted and did not accept chin contact, along with the mean ± standard error of the mean. The bars labeled with different letters indicate a significant difference based on the Tuckey’s test, *p* < 0.05, for attitude and response to observer, and the *t*-test for chin contact.

Table 5 shows that 82% (63 + 19%) of the kiln donkeys were apathetic or depressed, 68% (24 + 7 + 37%) were avoidant, ran away, or were aggressive, and 64% did not accept chin contact. To explore the association between body condition and behavior parameters, an ANOVA test was performed for the general attitude and reaction to an observer, and a t-test was performed for chin contact. A correlation was found between the general attitude and BCS (*p* < 0.01), reaction to observer and BCS (*p* < 0.01), and chin contact and BCS. Tuckey’s pairwise comparison test was used to compare the difference between behavior and reactions based on BCS (refer to Table 5). When measuring behavioral responses in donkeys there was a correlation between response and BCS. Donkeys that expressed an alert behavior had a BCS of 2.95 ± 0.14. A correlation between BCS and response to observers was found by donkeys being more reactive to observers had a higher BCS of 3.42 ± 0.14. A correlation was found between chin contact acceptance and BCS. The donkeys that allowed chin contact had a higher BCS (2.68 ± 0.11), compared to donkeys that did not accept chin contact (2.27 ± 0.07). Figure 7 shows the body condition means of the statuses of the behavior parameters along with the standard error and shows the groups that are significantly different based on Tuckey’s multiple comparisons as well.

The results suggest that the higher the BCS, the better the behavior of the donkey. We found an association between donkeys being injured and BCS, suggesting that body condition has an impact on the physical and behavioral conditions of working donkeys. Last, a correlation between donkeys with wounds from excessive force/beating and behavior was found (*p*-value < 0.05).

### 3.3. Physical Environmental Parameters

To explore the significant environmental parameters that have impacted the welfare of donkeys in brick kilns, including the BC, a multiple linear regression model was performed, with body condition as a response variable and all other environmental parameters included as explanatory variables. Table 6 shows the regression results and a numerical summary of the significant parameters. It was found that BCS was affected by three significant parameters (*p*-value < 0.05): hours worked/day, the number of donkeys working/brick kiln, and the cleanliness of the road. These three variables were able to explain 78.85% (R^2^ = 78.85%) of the variability seen in the body conditions. However, no significant difference was seen in the following parameters with BCS: production of bricks, which relates to how the drivers (children) are paid (this could have led to an increase in the pressure or beatings imposed on the donkeys), and/or the number of ovens/kilns.

As for the housing condition, we found that 65 ± 0.11% of the working donkeys drink salty or/and unclean water. The results suggest that the water condition has a significant impact on the body condition (*p*-value < 0.01). Another housing factor found that 85 ± 0.08% of working donkeys live in areas where there is mud, urine, and fecal matter on the floor of the donkey corrals/paddocks/stalls. Most of the donkeys’ housing (70 ± 0.10%) had poor ventilation and few windows. The floor conditions and ventilation have a significant impact on the body condition (the *p*-values are 0.011 and 0.000, respectively).

## 4. Discussion

Based on an ESPWWA estimation, there are more than 1000 brick kilns in Egypt, most of which are in the Giza and Qalyubia governorates. These brick kilns are not similar; they are different in terms of the mechanization, types of working equids, responsibilities of the stakeholders, and ownership of the donkeys. In the El-Saf kilns, the ownership of the donkeys belongs to the brick kiln owners, and the donkey drivers are children or adolescents, who are usually illiterate and do not have enough knowledge to properly communicate with the working equids. They live in villages near the brick kilns, and most of them are relatives of the owners or foreman. Working in these brick kilns is a temporary job for them. There is a dynamic movement of the handlers from kilns to other locations, and all stakeholders in the brick kilns are under pressure to work in order to meet the daily brick production target, even in harsh working conditions and bad weather.

Wounds or lesions have been considered to be one of the most prevalent and severe welfare problems facing working donkeys [3,4,5,23,24]. Wounds and lesions may have many origins, but little research has focused on the cause of wounds and their relationship to specific welfare parameters. This is true for donkeys working in brick kilns in various locations in Egypt. Some of the expected causes of wounds are improperly fitted and designed harnesses (saddles, collars), poor materials used to make the harness (natural and synthetic), poor communication between handlers (beating wounds) and their donkeys, aggressive behavior from other donkeys (e.g., bites and kicks), and improper husbandry/management practices [4,5].

This study revealed that the overall wound prevalence in working donkeys in brick kilns was high (80%), and it is by far the highest prevalence recorded, compared with other studies. For example, 77.5% was reported by Curran et al. [8], 79.4% was reported by Biffa and Woldemeskel [23], 48.9% was reported by Moltumo et al. [24], 42.2% was reported by Birhan et al. [25], 54.9% was reported by Fikru et al. [26], 59% was reported by Burn et al. [27] in Jordan, and 54% was reported by Sells et al. [28] in Morocco. This difference in the prevalence is attributed to the difference in environmental conditions, type of work, and the harness system for the donkeys in brick kilns. Brick kiln donkeys have the highest prevalence of the most severe lesions [5], which lead to severe pain and chronic suffering [29].

They often receive inadequate nutrition and veterinary care; they are overworked and overloaded; and they wear poorly fitted, insufficiently padded harnesses and are hitched to poorly designed harness [4], which may lead to many welfare problems, including harness-induced injuries, dehydration, and beating wounds [25]. In the El-Saf brick kilns, there is no training for donkeys to do such hard work, where sometimes they pull brick loads weighing 6 to 12 times their body weight. The work is often prolonged and strenuous under high environmental temperatures, with heat radiating from the kilns themselves. The terrain is harsh, there is limited access to clean fresh water, and the donkeys receive relatively little husbandry or veterinary care. There is a large proportion of working donkeys that are suffering from injuries due to poor handling by their handlers [29,30].

This study revealed that 49% of donkeys have wounds from excessive force or from being beaten, which is considered to be the most serious wound for donkeys. Pritchard et al. [3] reported the prevalence of hindquarter wounds from mistreatment to be 12.1% in donkeys. Another study [25] reported the prevalence of lesions from mistreatment to be 25.2% in donkeys and 41.7% in mules. The difference reported by other researchers and what was found in this study is likely due to the overall health of the working equids, the species (donkey or mule), the type of work, and environmental conditions. Considering that most drivers in El-Saf kilns are children or adolescents with little knowledge or skills relating to how to communicate and care for donkeys, coupled with their poor attitude toward donkeys, it is no surprise that the donkeys are mistreated. The drivers have little to no motivation to enhance the welfare of these equids, and so we find donkeys to be the most neglected animal. Brick kiln owners consider donkeys as machines with a low economic value, and the stakeholders of brick kilns work under severe pressure to meet daily production targets. From personal observations, it was frequently observed that the foreman in the El-Saf kilns imposed additional pressure on the young handlers, which resulted in the children beating their donkeys to achieve production goals.

A decrease in injuries may have been recorded if properly designed, well-fitted, and comfortable harnesses had been used in the brick kilns, or if the drivers had access to training relating to how to construct such harnesses. We know from other studies that properly fitted harnesses allow working animals to pull the equipment to the best of their ability, without risk of injuries. Unfortunately, we found many harnesses that were poorly designed or ill-fitted, which relates to the inefficient transfer of power from the animal to the implement, fatigue, discomfort, and/or injury to the animal [29]. While harness wounds can be avoided and represent 70% of veterinary intervention for working equids in developing countries, similar conditions were observed in this study. We could improve the performance of working equids by improving the overall harness quality and fit, as well as the material used to make the harnesses. With such improvements, we would likely see a decrease in wounds in the El-Saf brick kilns, as reported in other studies [31].

There are no available data on the harnesses of working donkeys in Egyptian brick kilns. This study revealed the prevalence of wounds caused by harness in working donkeys in brick kilns was correlated with certain parts of the harness such as the pack saddle, neck collar, and breeching or the wound was caused by the shafts of the cart. It is difficult to compare the prevalence of harness wounds found in this study with that found in other studies due to the fact that the harness system used in Egyptian brick kilns is different from that used in other studied areas, such as Ethiopia and Morocco [32]. Donkeys working in the El-Saf brick kilns are found pulling overloaded carts/wagons with harnesses in poor condition and ill-fitting and the cart is poorly attached to the donkey (Figure 5).

The study showed that the frequency of the wounds found on the limbs were related to the harness quality (e.g., poor) and donkeys being overworked and overloaded. This study and a study by Pearson et al. [29] showed that the level of severity and location of the wounds was associated with parts of the harness, BCS, and/or mistreatment.

This study revealed a correlation between cleanliness and wounds in the following locations and parts of the harness: the neck collar, pack saddle, and breeching. In 2009, Sells et al. [28] found that the cleanliness of the pack saddles is a significant factor in developing a pack wound. The presence of dirt may contribute to the early stages of wound formation by increasing the abrasiveness of the material. A poorly designed or ill-fitted harness will result in fatigue, discomfort, or lesions on donkeys [33].

We found that there is an association between beating wounds and saddle and breeching wounds (*p*-value < 0.01). In addition, the association between beating wounds and the cleanliness, condition, and fit of different parts of the harness was explored, and it was found that it is associated with the cleanliness of the saddle and breeching and associated with the fitness of the neck collar as well. Moreover, because the poorly designed or ill-fitted and unclean harness leads to an inefficient transfer of power from the donkey to the cart [33], painful lesions, which reduce the work capacity of working donkeys [34], it will result in an increase in the frequency of being beaten by their handlers to meet the daily production target.

Regarding the BCS of working donkeys in brick kilns, it is found that BCS is an important animal-based indicator in assessing the health status and welfare of working equids [35,36], without determining whether the nutrient requirements are met or exceeded [37]. A healthy equid should be fit, neither not too fat nor too thin [38]. A poor body condition is a major welfare concern for working equids [5,39]. Thin donkeys have less natural padding (adipose tissue and muscle), which protects them from friction, pressure, and lesions caused by harnessing. The present study revealed that 56% of kiln donkeys have a BCS of 2 or less. This score is higher than what other studies have reported for donkeys in similar scenarios [24,25]. This difference may be attributed to a difference in the working and environmental conditions, management practice, working load, and type of work. In general, poor body condition scores of working equids may be attributed to malnutrition [40] and/or parasitic infestation, coupled with a heavy working load [41]. Working equids used for strenuous work in hotter weather and or hot and humid weather are likely to suffer from dehydration and a poor body condition [42].

A poor body condition is generally coupled with many underlying factors, such as exhaustion from being overworked [30] and chronic pain [43]. Donkeys in such conditions may suffer from negative mental states (e.g., depression) [44] and live a life that is generally characterized by poor welfare [45,46,47].

The study found a difference between injured donkeys and healthy donkeys in terms of BCS. This finding suggests that a higher BCS and likely an overall improved care may decrease the number and severity of wounds. Pritchard et al. [3] identified the highest correlation coefficient to be that between a low body condition score and wounds of the skin and deeper tissues (r = 0.37), because they may have less natural padding protecting them from pressure, friction and shear lesions caused by the harness. The same results were reported by Abdela et al. [40], who found that there was a significant association between body condition and occurrences of wounds (*p*-value = 0.000), and animals with a poor body condition are more likely to be wounded than animals found to be in a good body condition. This finding agrees with the report of Birhan et al. [25] and Tsega et al. [41], who reported a significant difference between a poor and good BCS.

Behavior can be modified by several variables, including the following: workload, working conditions, and housing environment, as reported in this study. There is a need to understand the behavior of working donkeys and how it is associated with different management and production demands in order to improve the conditions and welfare of donkeys. Behavioral tests, such as those used in this study, can begin to identify compromised behavior that is linked to poor welfare, from a low BCS to excessive injuries or animals that have become apathetic due to being overworked.

In the present study, the majority of the donkeys displayed behavioral signs associated with being apathetic or depressed, and some even showed signs of aggression. The results in our study, compared to other studies, such as that of Ali et al. [18], suggest the findings that apathetic, depression and aggressive behaviors are more prevalent. Pritchard et al. [35] suggested that behavior could be related to or attributed to the severity of work, environmental and working conditions, type of work, and prevalence of harness-induced wounds in donkeys. The results in this study showed that donkeys had multiple challenges to overcome in order to live a healthy life. Factors that influenced their welfare and were documented included poor nutrition, water sources, harnesses, mental states, and overall BCS, which led to a compromised welfare. Such parameters have been shown to have similar results in other working equid studies [4]. Brick kiln donkeys have the highest prevalence of the most severe lesions [42]. In the El-Saf brick kilns, as mentioned before, the prevalence of wounds was the highest, compared with previous reports, and the prevalence of beating wounds in the present study was higher than the prevalence of mistreatment lesions reported by Ali et al. [18]. Burn et al. [4] reported that working equids that suffer from severe physical injuries showed depression and unresponsive behavior. Unresponsiveness behavior is considered to be an indicator of several poor welfare problems, for example, fatigue due to a heavy workload [30,48] and chronic pain [30] in the El-Saf brick kiln. The donkeys and their handlers are generally found to work for more than 8 h without rest in extreme conditions that are hot and to be under a high production pressure. Donkeys are generally overloaded and pull carts averaging 2.25 tons, in addition to the weight of the handlers, while suffering from pain and open lesions. In many cases, we found donkeys displaying aggressive reactions toward observers due to the working conditions and the fact the donkeys were regularly beaten by their handlers. This resulted in continuously compromised human–donkey interactions, and the donkeys continued to develop behaviors, such as fearfulness and aggression toward humans, which is generally not commonly observed in donkeys [42]. Rousing et al. [43] reported that depressed and unresponsive donkeys experienced improper handling. Positive human–animal interactions are very important when it comes to working equids, as it facilitates daily activities, encourages positive human attitudes, and improves animal welfare [44]. Burn et al. [4] reported that a low BCS is correlated most strongly with unresponsive behavior. The causes of a low BCS are multifactorial and likely include malnutrition, overwork, parasitism, and disease, which can simultaneously cause behavioral sickness that leads to anorexia and the development of a poor BCS [38,44,45]. Both fatigue from overworking, being overloaded [16] coupled with chronic pain [30], and depression or learned helplessness [36] can lead to a compromised welfare [37,38,39]. In the present study, we found that donkeys with a higher BCS show more alert behaviors and are friendlier with humans.

## 5. Conclusions

There is a need for basic research to understand the management, environmental, and working conditions in brick kilns to be able to promote a sustainable improvement program for donkey welfare. The El-Saf brick kilns are similar in terms of the work and mechanization systems, but there are differences in terms of the management practices and resources available for the donkeys in each kiln. This study introduced methods to measure the welfare status of the working donkeys in the El-Saf brick kilns and how to identify associated risk factors that compromise their welfare. We have found that the variation in the level of welfare associated with the donkeys in each kiln, along with differences found in the management practices, can help us to better understand why some kilns have a better welfare than others. The behavior, along with the welfare, of many of the donkeys was found to be compromised. The highest incidence of wounds was caused by beating, improper handling, and/or poor communication, which all reflected the missing link in human–animal interactions. Future studies could possibly measure the attitudes of donkey handlers in relation to overall donkey welfare. Another possibility would be measuring the correlation of human welfare to donkey welfare in each kiln. We believe that increasing brick kiln managers’ and workers’ knowledge of proper donkey husbandry, care, management, harness design, and function, as well as providing them with information on working donkeys, could improve the overall welfare of working donkeys. A standard protocol would need to be developed and shared in training sessions with brick kiln managers and then handlers. A further investigation may be warranted to understand the attitudes and knowledge of donkey handlers concerning the donkeys in brick kilns. Then, a strategy showing that an improved welfare (e.g., a higher BCS) leads to fewer wounds or clean water and improves the production of the donkeys and handlers is essential. This would be key to gaining the assent of stakeholders. A step toward this goal is understanding the risk factors, which this study has accomplished. We can now a design an effective educational program for the El-Saf brick kilns, which can be applied to all Egyptian Brick kilns. This study suggests that improvements can easily be made through knowledge transfer, if human behavior change can be facilitated.

## Figures and Tables

**Table 1:** The harness condition scores.

Grade	Description
1 = very bad	Poor contact surfaces due to excessive wear, broken parts, or bad construction (despite being new), and the use of non-equine-friendly materials, such as wire, plastics, and nylon, in areas that are in physical contact with the donkey
2 = bad	Well used, but developing problems with contact surfaces
3 = fair	Well used, but in a good condition and with good contact surfaces for the donkey
4 = good	A harness level that falls between 3 and 5
5 = very good	Well made, not well used, with good contact surfaces for the donkey

**Table 2:** Harness cleanliness scores.

Grade	Description
1 = bad	Encrusted and dried on bodies (blood, mud, feces, etc.) and likely to cause injury
2 = fair	Dusty, but not abrasive
3 = good	Totally dirt free

**Table 3:** Cart condition scores.

Grade	Description
1 = bad	Very poor, with no bearing left, and the shaft is narrow, causing injuries to donkeys’ sides. It may contain sharp objects, and the tires are deflated
2 = fair	The wheel bearings have space to move from one side to the other, the shaft is tight, and the tires are not completely deflated, but the pressue inside is low
3 = good	The wheel bearings have no space to move from side to side, the wheels easily rotate, the distance between the two shafts is wide enough to prevent injuries to the donkeys’ sides, and the tires are not deflated

**Table 4:** Numerical summary of the area of different wound types (cm^2^).

Wound Type	Mean ± SE ^1^	Median	Minimum	Maximum
Saddle	25.86 ± 3.60	13.00	0.13	150.0
Beating	47.95 ± 6.26	30.00	1.75	365.5
Limb	35.5 ± 17.80	8.50	0.20	385.0
Neck	23.32 ± 4.75	7.50	0.25	197.5
Breeching	77.80 ± 40.3	19.10	0.80	625.0
Shaft	89.60 ± 31.2	16.80	0.30	577.0

^1^ SE = standard error of the mean.

**Table 5:** Numerical summary of body condition for different statuses of general attitude, reaction to an observer, and chin contact, along with the statistical test values and *p*-values of the t-test and ANOVA test.

Parameter	Donkey Status	N (%)	Mean ± SE ^1^	Test Value	*p*-Value
General attitude	Alert	32 (18%)	2.95 ± 0.14 ^a^	F-value = 10.38	<0.001 **
	Apathetic	113 (63%)	2.34 ± 0.07 ^b^		
	Depressed	34 (19%)	2.14 ± 0.14 ^b^		
Reaction to observer	Discovering	13 (7%)	3.42 ± 0.14 ^a^	F-value = 6.65	<0.001 **
	Not moving	44 (25%)	2.46 ± 0.13 ^b^		
	Avoiding	43 (24%)	2.35 ± 0.12 ^b^		
	Running away	13 (7%)	2.35 ± 0.17 ^b^		
	Being aggressive	66 (37%)	2.24 ± 0.09 ^b^		
Chin contact	Accepting	64 (36%)	2.68 ± 0.11 ^a^	T-value = 3.24	0.002 **
	Not accepting	115 (64%)	2.27 ± 0.07 ^b^		

^1^ SE = standard error of the mean, ** means significant at the 1% significance level, shared letters are not significantly different groups, based on the Tuckey’s multiple comparison method. ^a^ refers to significant different status compared to the others and ^b^ refers to nonsignificant values in each group.

**Table 6:** Numerical summary of the environmental parameters (median and mean ± standard error), and the coefficient values, t-values, and *p*-values of the multiple linear regression.

Parameter	Mean ± SE ^1^	Median	Coef ^2^	T-Value	*p*-Value
Working hours/day	6.23 ± 0.19	6.0	−0.32	−2.75	0.016 *
Number of donkeys	8.95 ± 0.60	7.5	0.22	4.20	0.001 **
Distance	54.70 ± 3.53	53.5	−0.02	−2.40	0.031 *
Concrete amount/donkey	2.52 ± 0.20	2.15	0.48	4.85	0.000 **

^1^ SE = standard error of the mean, * means significant at the 5% significance level, and ** means significant at the 1% significance level, ^2^ Coef = coefficient value
